# IFN‐γ^+^IL‐17^+^Th17 cells regulate fibrosis through secreting IL‐21 in systemic scleroderma

**DOI:** 10.1111/jcmm.15266

**Published:** 2020-11-06

**Authors:** Xiaojing Xing, Anqi Li, Hong Tan, Yong Zhou

**Affiliations:** ^1^ Department of Dermatology First Medical Centre of Chinese PLA General Hospital Beijing China; ^2^ Medical College of Nankai University Tianjin China

**Keywords:** cytokines, IFN‐γ, IL‐17, IL‐21, systemic scleroderma, Th 17 cells

## Abstract

This study aimed to explore the function of IFN‐γ^+^IL‐17^+^Th17 cells on fibrosis in systemic scleroderma (SSc). Blood and skin samples were collected from 20 SSc cases and 10 healthy individuals. The percentage of IFN‐γ^+^IL‐17^+^Th17 cells was detected using flow cytometry. The in vitro induction of IFN‐γ^+^IL‐17^+^Th17 cells was performed adopting PHA and rIL‐12. Gene expression was detected via quantitative real‐time polymerase chain reaction (qRT‐PCR), whereas western blot analysis was adopted for protein analysis. The distribution of IFN‐γ^+^IL‐17^+^Th17 cells was significantly increased in SSc cases and positively correlated with SSc stages (*P* = .031), disease duration (*P* = .016), activity (*P* = .025) and skin scores (*P* < .001). In vitro, IFN‐γ^+^IL‐17^+^Th17 cells could promote the expressions of α‐SMA and COL1A1, revealing increased fibroblasts’ proliferation and enhanced collagen‐secreting capacity. In addition, IL‐21 expression was significantly increased in co‐culture medium of IFN‐γ^+^IL‐17^+^Th17 cells and fibroblasts (*P* < .001). IL‐21 neutralizer treatment resulted in the down‐regulation of α‐SMA and COL1A1. IL‐21 was confirmed as an effector of IFN‐γ^+^IL‐17^+^Th17 cells in fibrosis process. The distribution of IFN‐γ^+^IL‐17^+^Th17 cells was significantly increased in SSc cases and positively correlated with disease activity. IFN‐γ^+^IL‐17^+^Th17 cells could promote fibroblast proliferation and enhance collagen‐secreting ability via producing IL‐21, thus contributing to fibrosis in SSc.

## 1 INTRODUCTION

Systemic scleroderma (SSc) is a multisystem autoimmune disease and characterized by skin and visceral fibrosis, and vascular damages.^1^ SSc not only contributes to high mortality, but also causes various severe and disabling complications, such as arrhythmias, myocardial dysfunction and pulmonary arterial hypertension.^2^, ^3^ According to the extent of skin sclerosis, SSc can be divided into diffuse and limited subtypes.^4^ Until now, the pathogenesis of SSc has not been explained completely. SSc is considered as a complex result mediated by alterations in immune systems, and genetic and environmental factors.^5^

T helper cells play important roles in immunity and autoimmunity via secreting cytokine.^6^ The activation of T helper cells and excessive production of cytokines are closely correlated with SSc pathogenesis through enhancing fibrosis and inflammation.^7^ Th17 cells, a subgroup of T helper cells, belong to CD4^+^ T lymphocytes. The main function of Th17 cells is to recruit and activate neutrophils and induce pro‐inflammatory signal pathways.^8^ Th17 cells hold high plasticity. In addition to IL‐17, Th17 cells also secrete other cytokines under specific inflammation conditions, including TNF‐α, IFN‐γ, IL‐21 and IL‐22.^9^ Th17 cells are linked to multiple autoimmune diseases, such as psoriasis,^10^ inflammatory bowel diseases,^11^ rheumatoid arthritis^12^ and SSc.^13^ In mouse bleomycin (BLM) model of SSc, the percentages of Th17 cells were positively correlated with skin and lung inflammation scores and fibrosis severity.^14^ Th17 cells might promote the proliferation of fibroblasts and enhance cytokine production, thus contributing to fibrosis in SSc model.

IFN‐γ–producing Th17 cells (IFN‐γ^+^IL‐17^+^Th17 cells), also called Th17.1 cells, were originally identified in Crohn's disease, and they can produce both IL‐17 and IFN‐γ factors.^15^ IFN‐γ^+^IL‐17^+^Th17 cells are more pathogenic in autoimmune diseases, and their associations with autoimmune disorders have been reported in several articles. For example, Ramstein et al^16^ reported that the frequency of IFN‐γ^+^IL‐17^+^Th17 cells was high in sarcoidosis, and their percentage was higher than Th1‐cell percentages. In SSc, the levels of serum IL‐17 and IFN‐γ were significantly increased and showed close association with disease activity.^17^ However, whether IFN‐γ^+^IL‐17^+^Th17 cells were involved in the pathogenesis of SSc remained unclear.

In this study, we aimed to explore the distribution of IFN‐γ^+^IL‐17^+^Th17 cells in SSc cases and their association with disease severity. In addition, possible mechanisms of IFN‐γ^+^IL‐17^+^Th17 cells affecting SSc progression were also investigated.

## 2 MATERIALS AND METHODS

### 2.1 Study subjects

Our investigation protocols were approved by the Ethics Committee of First Medical Center of Chinese PLA General Hospital. All eligible participants signed written informed consents before sample collection.

Twenty eligible SSc patients were consecutive dermatological outpatients or inpatients of our hospital. SSc patients were diagnosed based on the classification criteria for SSc proposed by the American College of Rheumatology in 2013.^18^ Based on lesion and clinical symptoms, patients with other skin diseases, such as contact dermatitis and psoriasis, were excluded from our study. In addition, 10 healthy individuals were recruited from the Medical Center of our hospital and employed as healthy controls. The healthy individuals had no history of skin diseases. The cases and controls were matched in age and gender. According to disease history, clinical symptoms and pathological changes in skin, the patients were dived into early and non‐early stages.^18^ Patients in early stage group experienced no treatment of cyclophosphamide shock or methotrexate. If patients had been treated by prednisone, the maximum dose was less than 30 mg/day. Moreover, their disease duration was less than 5 years. For patients in non‐early stage group, the above‐mentioned limitations were not applied. The basic features of the patients were collected from their medical records. The disease activity of the patients was estimated according to European Scleroderma Study Group disease activity score for SSc (Valentini disease activity index).^19^ Skin involvement among the patients was estimated using the modified Rodnan skin score.^20^

### 2.2 Specimens

Full‐thickness skin biopsy specimens (3 cm) were obtained from diseased lesions for the patients. In addition, site‐matched normal skin tissues were also obtained from the healthy controls. Then, the obtained skin samples were embedded in paraffin for immunohistochemical analysis.

Peripheral blood samples (5 mL) were collected from both of SSc cases and healthy individuals. Then, the blood samples were diluted using phosphate buffer saline (PBS) and subjected to peripheral blood mononuclear cell (PBMC) isolation via the method of ficoll density‐gradient centrifugation. PBMCs were cultured using RPMI media (Sigma Chem. Co.) at 37°C with the presence of 5% CO_2_.

### 2.3 Dermal fibroblast isolation and culture

Dermal fibroblasts were isolated from skin biopsy according to the descriptions by Philippeos et al^21^ Isolated dermal fibroblasts were cultured using DMEM with 10% FBS. Cell medium was incubated in a humid atmosphere at 37°C with the presence of 5% CO_2_. The cell medium was changed every 3 or 5 days; when cell confluent reached 80%, cell passage was performed.

### 2.4 The induction of IFN‐γ^+^IL‐17^+^Th17 cells

Human naive CD4^+^ T cells were isolated from the obtained PBMCs using EasySep™ Human Naive CD4^+^ T Cell Isolation Kit and seeded to a 96‐well plate which contained 200 μL X‐VIVO15 (LONZA) medium, at a density of 2 × 10^4^ cells/mL. Then, the cells were stimulated utilizing 2.5 μg/mL polyhydroxyalkanoates (PHA) and rIL‐12 (50 ng/mL) (Sigma‐Aldrich). The cells were incubated for 7 days.

### 2.5 Co‐culture of IFN‐γ^+^IL‐17^+^Th17 cells and dermal fibroblasts

Dermal fibroblasts were cultured in 48‐well plate using complete cell medium, and kept serum starvation for 12 hours prior to co‐culture. Then, dermal fibroblasts were co‐cultured with induced IFN‐γ^+^IL‐17^+^Th17 cells (1:2 dilution) for 3 days. The cells were harvested for subsequent detection. Dermal fibroblasts cultured with CD4^+^ T cells without stimulation were adopted as controls. In addition, for IL‐21 inhibition experiments, the cultured dermal fibroblasts were pre‐incubated using neutralizing antibody (0.1 µg/mL, PeproTech) for 1 hour, and then, co‐culture experiments were performed.

### 2.6 Flow cytometric analyses

To determinate the frequency of IFN‐γ^+^IL‐17^+^Th17 cells in peripheral blood, flow cytometric analyses were performed. At first, the cells were prepared for cellular cytokine staining. 1 × 10^6^ cells/mL PBMCs were cultured and incubated with phytohaemagglutinin (PHA) (10 μg/mL) for 10 hours at 37°C with 5% CO_2_. Then, brefeldin A (BFA; eBioscience) (3 μg/mL) with 1 μg/mL GolgiPlug™ (Becton Dickinson Biosciences) was added and incubated for additional 8 hours to stop cytokine production. Subsequently, the cells were washed using PBS with 1% bovine serum albumin and suspended in 0.25 mL of cold 4% PFA fixative. Then, the cells were stained by anti‐human PerCP‐conjugated IFN‐γ and PE‐conjugated anti‐human IL‐17 antibody, which was performed using Fixation/Permeabilization Staining Kit (BD Biosciences) following the instructions of the manufacturer. Later, staining results were analysed employing FACSCalibur instrument with CellQuest software (BD Biosciences). WinMDI 2.8 software (Joseph Trotter, Scripps Clinic) was adopted for data analysis.

### 2.7 Immunohistochemistry assay

Formalin‐fixed skin tissues were cut into 4 μm‐thick sections for histological examinations. At first, the tissue sections were incubated with 3% H_2_O_2_ in absolute methanol to quench endogenous peroxidase. After washed three times adopting PBS, the sections were blocked using 2% goat serum. Then, the sections were incubated with specific primary antibodies overnight at 4°C, and adopted antibodies were as follows: anti‐IFN‐γ antibody (1:100, Abcam) and anti‐IL‐17 antibody (1:50, Abcam). Subsequently, the sections were washed three times employing PBS and incubated with rabbit secondary antibodies for 2 hours at room temperature. Later, the sections were stained through avidin‐biotin method using diaminobenzidine. Five high power microscopes were adopted for each tissue specimen, and the percentages of positive cells were automatically analysed by Image‐Pro Plus 6.0 software (Media cybernetics). The positivity was defined as the percentages of positively stained cells in all cells for each high power microscope, and average value for five randomly selected views was calculated for final positivity.

### 2.8 Quantitative real‐time polymerase chain reaction (qRT‐PCR)

Total RNA was extracted from cells using TRIzol reagent (Invitrogen), and its quality was estimated utilizing a NanoDrop 2000 ultraviolet spectrophotometer (Thermo Scientific). 5 μg RNA was synthesized into the first chain of cDNA, which was performed using PrimerScript RT reagent kit (Takara). Then, qRT‐PCR was carried out using SYBR Green PCR Master Mix (Applied Biosystems) on 7300 Real‐Time PCR System (Applied Biosystems). GAPDH was employed as internal control. The used primer sequences were as follows: IL‐17 forward 5′‐GAAGGCAGGAATCACAATCCC‐3′, reverse 5′‐GCTGTCTTTGAAGGATGAG‐3′; IFN‐γ forward: 5′‐CAGATGTAGCGGATAATGGA‐3′, reverse: 5′‐TCACTTGGATGAGTTCATGT‐3′; IL‐21: forward: 5′‐GGAGAAGACAGAAACACAGACTAAC‐3′, reverse: 5′‐CTAGAGGACAGATGCTGATGAATC‐3′; COL1A1 forward 5′‐CCTGGAAAGAATGGAGATGATG‐3′, reverse 5′‐ATCCAAACCACTGAAACCTCTG‐3′; α‐SMA forward 5′‐CTATGAGGGCTATGCCTTGCC‐3′, reverse 5′‐GCTCAGCAGTAGTAACGAAGGA‐3′; GAPDH forward 5′‐GGTGAAGGTCGGTGTGAACG‐3′, reverse 5′‐CTCGCTCCTGGAAGATGGTG‐3′. Relative expressions of targeted mRNAs were normalized to GAPDH and analysed using the equation of 2^−ΔΔCt^. Each sample was tested three times.

### 2.9 Western blot

Protein samples were isolated from harvested cells using 500 μL radio‐immunoprecipitation assay (RIPA) buffer (Thermo Scientific) with 1 mmol/L phenylmethane sulfonyl fluoride. Then, protein samples were obtained through centrifugation. Protein samples of same volume were subjected to 10% sodium dodecyl sulphate‐polyacrylamide gel electrophoresis and transferred to polyvinylidene fluoride membrane (PVDF) (0.45 µm pore size; EMD Millipore). Later, the membranes were blocked by 5% skimmed milk and incubated with primary antibodies at 4°C overnight. GAPDH acted as loading control. Then, the membranes were incubated employing secondary antibody at room temperature for additional 2 hours. Blotting results were analysed applying an ECL blotting analysis system (GE Healthcare Life Sciences). Each trial was repeated at least three times.

### 2.10 Statistical analysis

All data analyses were performed using SPSS 18.0 software (SPSS, Inc), and GraphPad Prism version 5.0 (GraphPad) was adopted for figure plotting. The distribution of continuous variables was estimated using the method of Kolmogorov‐Smirnov test. If data were in normal distribution, their comparison between two groups was performed via Student's t test; otherwise, Mann‐Whitney U test was adopted. Continuous data were shown by mean ± standard deviation (SD), median and range. Differences in classified variables between groups were analysed using chi‐square test. In addition, correlation analysis was performed through Pearson correlation method. *P *< .05 indicated statistical significance of the results.

## 3 RESULTS

### 3.1 Baseline characteristics of the study participants

There were 20 SSc patients and 10 healthy individuals in our study. The SSc group included 11 males and 9 females, whose average age was 56.15 ± 12.02 years. There were 5 men and 5 women in healthy control group, with an average age of 53.60 ± 12.04 years. The case and control groups were matched in age (*P* = .308) and gender (*P* = .883). In SSc group, 9 (45%) cases were confirmed with limited SSc, and the rest 11 (55%) ones with diffuse SSc. Their average disease duration was 3.40 ± 1.60 years, and their mean Valentini score was 2.35 ± 1.53. The mean mRSS (modified Rodnan skin score) was 8.40 ± 5.65. Pulmonary involvement was observed in 5 (25%) patients and digital necrosis in 10 (50%) cases, while renal involvement in 3 (6%) cases. Autoantibody detection results demonstrated that 15 (75%) cases were positive to anti‐nuclear antibody, while 4(20%) positive to anti‐centromere antibody. 11 SSc patients were categorized into early stages and 9 into non‐early stage groups. Comparison analysis demonstrated significant differences in disease duration (*P < *.001), disease activity (*P* = .006), mRSS (*P < *.001), digital necrosis (*P* = .002) and renal involvement (*P* = .038) between patients at early and non‐early stages. Detailed characteristics of SSc patients were summarized in Table 1.

**TABLE 1:** Characteristics of SSc patients

Characteristics	Total (n = 20)	Early stages (n = 11)	Non‐early stages (n = 9)	*P* values
Age (years)	56.15 ± 12.02	57.09 ± 12.09	55.00 ± 12.56	.710
Median (range)	53.5 (37‐75)	60 (37‐72)	53.5 (40‐75)	
Gender				.391
Male	11 (55%)	7 (63.64%)	4 (44.44%)	
Female	9 (45%)	4 (36.36%)	5 (55.56%)	
Subtypes of SSc				.343
Limited SSc	9(45%)	6 (54.55%)	3 (33.33%)	
Diffuse SSc	11 (55%)	5 (45.45%)	6 (66.67%)	
Duration of disease (years)	3.40 ± 1.60	2.36 ± 0.92	4.67 ± 11.32	<.001
Median (range)	3 (1‐7)	2 (1‐4)	1.5 (3‐7)	
Disease activity	2.35 ± 1.53	1.55 ± 0.93	3.33 ± 1.58	.006
Median (range)	2 (0‐6)	1 (0‐3)	3 (1‐6)	
Pulmonary involvement	5 (25%)	1 (9.1%)	4 (44.44%)	.069
Digital necrosis	10 (50%)	2 (18.18%)	8 (88.89%)	.02
Renal involvement	3 (6%)	0 (0%)	3 (33.33%)	.038
Autoantibodies				
Anti‐nuclear antibody	15 (75%)	8 (72.73%)	7 (77.78%)	.795
Anti‐centromere antibody	4 (20%)	2 (18.18%)	2 (22.22%)	.822
mRSS	8.40 ± 5.65	4.36 ± 2.62	13.33 ± 4.18	<.001
Median (range)	7.00 (1‐20)	4.0 (1‐10)	13.0 (7‐20)	

Abbreviation: mRSS, modified Rodnan skin score.

### 3.2 The frequency of IFN‐γ^+^IL‐17^+^Th17 cells was increased in SSc patients

**FIGURE 1:**
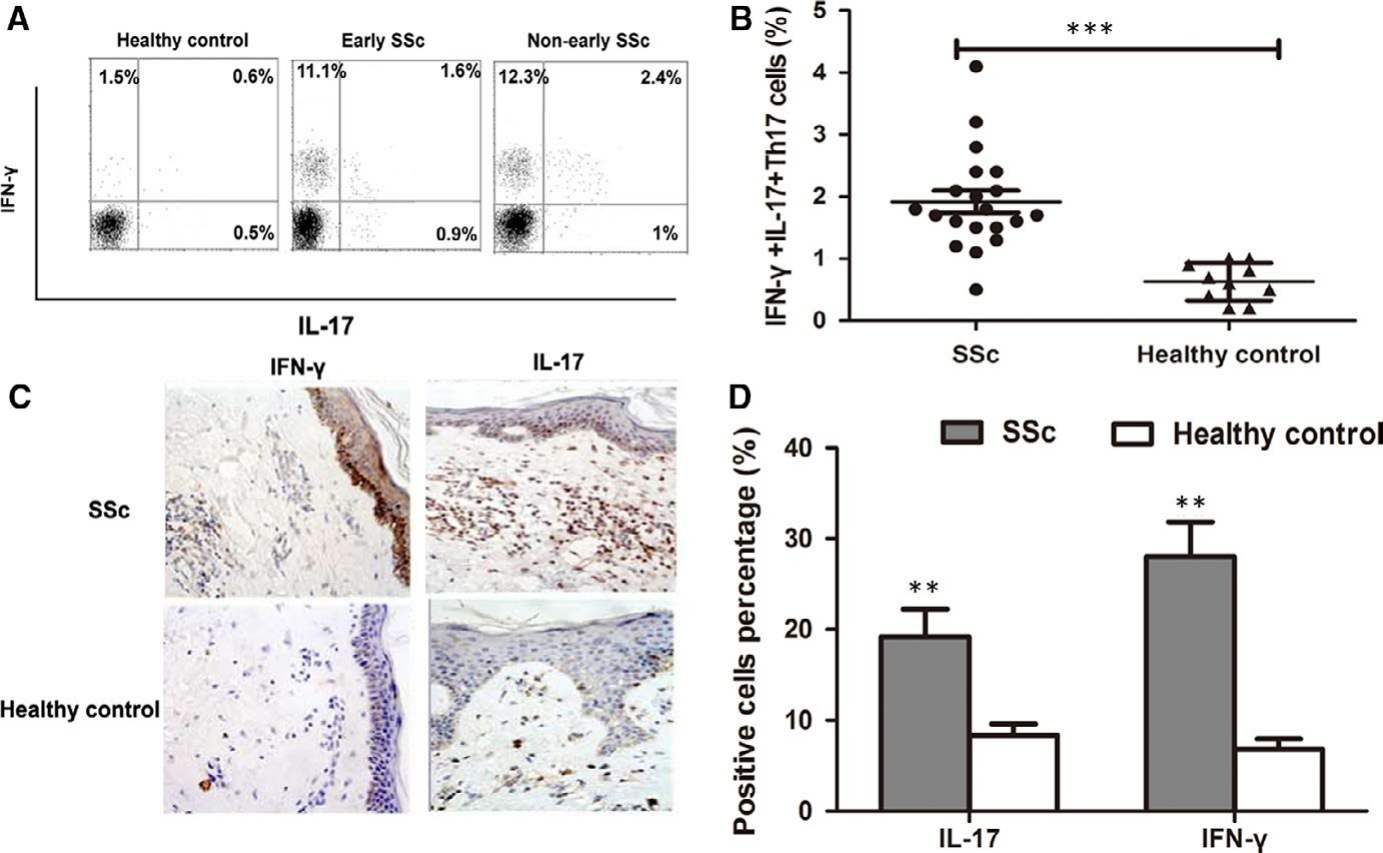
Representative flow cytometric assay for IFN‐γ^+^IL‐17^+^Th17 cell distributions in peripheral blood for SSc cases and healthy individuals (A). The frequency of IFN‐γ^+^IL‐17^+^Th17 cells was significantly higher in SSc cases than in healthy individuals (B). Representative immunohistochemistry staining images for IL‐17–positive and IFN‐γ–positive cells in SSc skin specimens and healthy controls × 250 (C). The percentages of positively stained cells of IFN‐γ and IL‐17 were significantly higher in SSc specimens than in healthy ones (D). **P < .01, ***P < .001

The frequency of IFN‐γ^+^IL‐17^+^Th17 cells in peripheral blood was detected for SSc cases and healthy individuals using flow cytometric assay (Figure 1A). Analysis results demonstrated that compared to healthy individuals, SSc patients showed significantly heightened frequency of IFN‐γ^+^IL‐17^+^Th17 cells (*P* < .001) (Figure 1B). In addition, immunohistochemistry assay was adopted to analyse the number of cells which were positive to IFN‐γ and IL‐17 staining in collected skin tissues (Figure 1C). The percentages of positively stained cells of IFN‐γ and IL‐17 were significantly higher in SSc specimens than in healthy ones (*P* < .01) (Figure 1D).

### 3.3 The mRNA levels of IFN‐γ and IL‐17 were significantly increased in SSc

**FIGURE 2:**
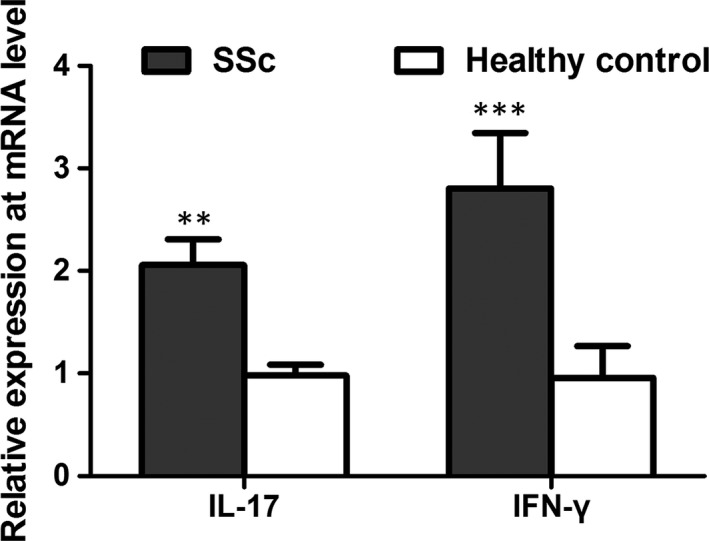
QRT‐PCR analysis suggested that the mRNA levels of IFN‐γ and IL‐17 were obviously increased in SSc cases, compared to healthy controls. **P < .01, ***P < .001

QRT‐PCR was performed to investigate the expression of cytokines produced by IFN‐γ^+^IL‐17^+^Th17 cells in blood samples for SSc cases and healthy controls. The results suggested that in comparison with healthy individuals, the mRNA levels of IFN‐γ and IL‐17 were obviously increased in SSc cases (*P* < .01) (Figure 2).

### 3.4 Association of IFN‐γ^+^IL‐17^+^Th17 cell distributions with clinical characteristics of SSc

**FIGURE 3:**
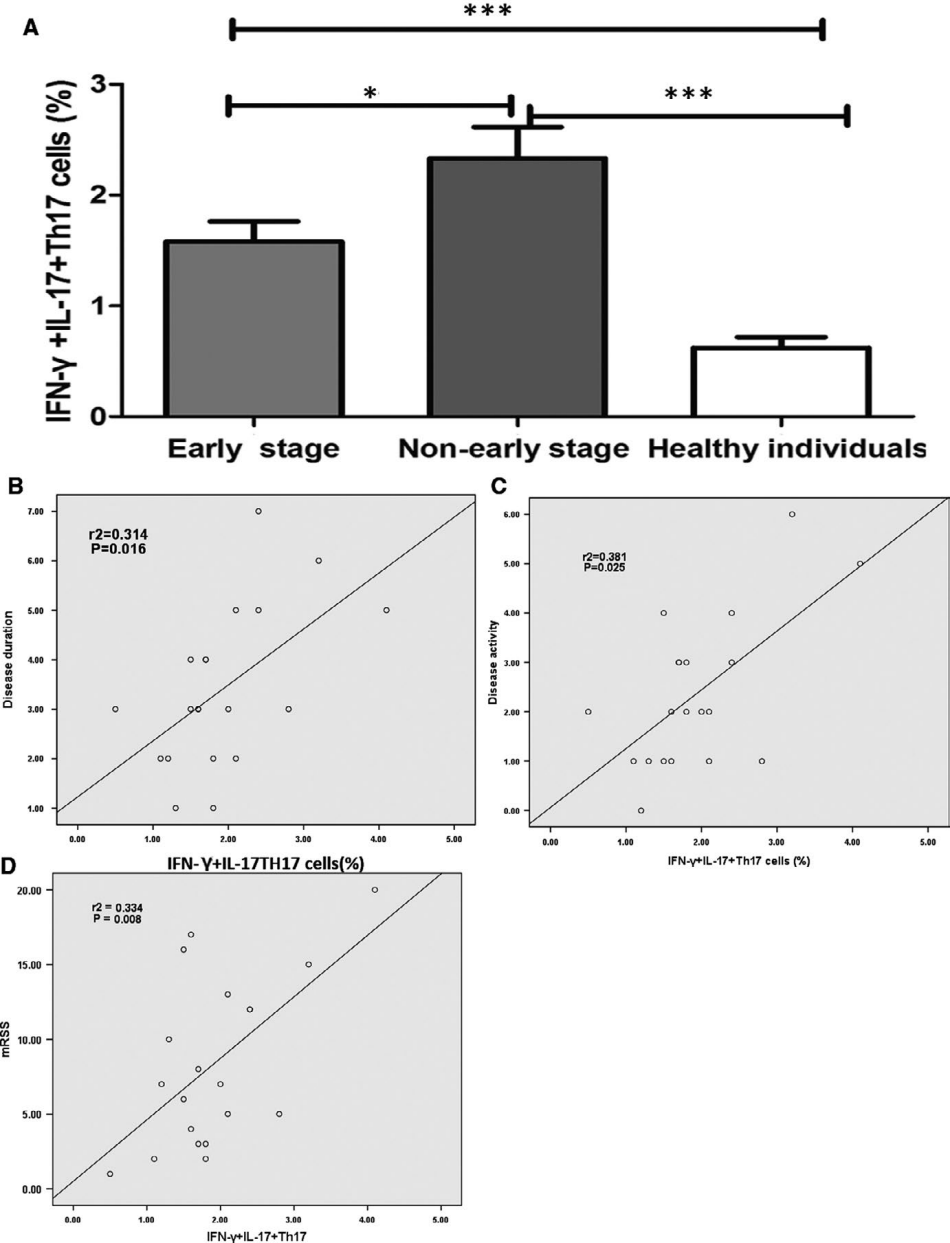
The distributions of IFN‐γ^+^IL‐17^+^Th17 cells were closely correlated with disease stages (A), duration (B), activity (C) and skin scores (D) in SSc cases. *P < .05, ***P < .001

The association of IFN‐γ^+^IL‐17^+^Th17 cells’ distributions with SSc severity was analysed in our study. We found that patients in non‐early stage group exhibited higher frequency of IFN‐γ^+^IL‐17^+^Th17 cells than those in early stages (*P* = .031). SSc patients in both groups showed increased frequency of IFN‐γ^+^IL‐17^+^Th17 cells compared to healthy individuals (*P* < .001 for both) (Figure 3A). In addition, correlation analyses were performed to investigate the association of IFN‐γ^+^IL‐17^+^Th17 cell distributions with disease duration, disease activity and mRSS. Relevant results demonstrated that the percentages of IFN‐γ^+^IL‐17^+^Th17 cells were positively correlated with disease duration (r^2^ = 0.314, *P* = .016), disease activity (r^2^ = 0.381, *P* = .025) and mRSS (r^2^ = 0.334, *P* = .008) (Figure 3B‐D).

### 3.5 In vitro induction promoted the differentiation towards IFN‐γ^+^IL‐17^+^Th17 cells

**FIGURE 4:**
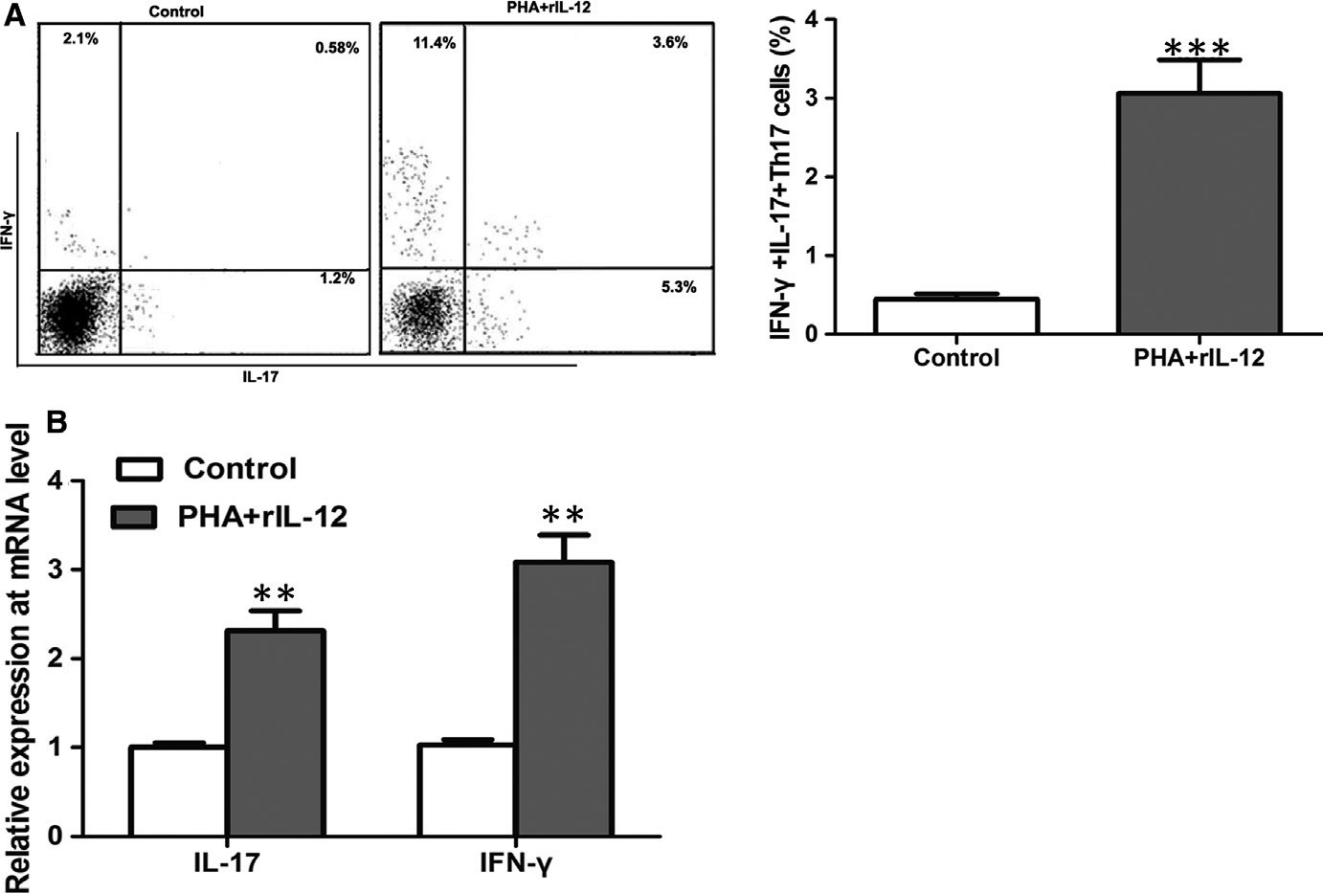
Flow cytometric analyses suggested that the induction of PHA and rIL‐12 promoted the percentage of IFN‐γ^+^IL‐17^+^Th17 cells in vitro (A). Moreover, the mRNA levels of IFN‐γ and IL‐17 were also remarkably increased (B). **P < .01, ***P < .001

In vitro experiments, PHA and rIL‐12 were applied to induce the differentiation of CD4^+^ T cells towards IFN‐γ^+^IL‐17^+^Th17 cells. Flow cytometric analyses suggested that after induction, the percentage of IFN‐γ^+^IL‐17^+^Th17 cells was significantly up‐regulated (*P* < .001) (Figure 4A). Moreover, the mRNA levels of IFN‐γ and IL‐17 were also remarkably increased (*P* < .01) (Figure 4B). All data revealed that PHA and rIL‐12 induction could promote T cells’ differentiation towards IFN‐γ^+^IL‐17^+^Th17 cells.

### 3.6 IFN‐γ^+^IL‐17^+^Th17 cells promoted dermal fibroblasts’ proliferation and their collagen‐secreting capacity

**FIGURE 5:**
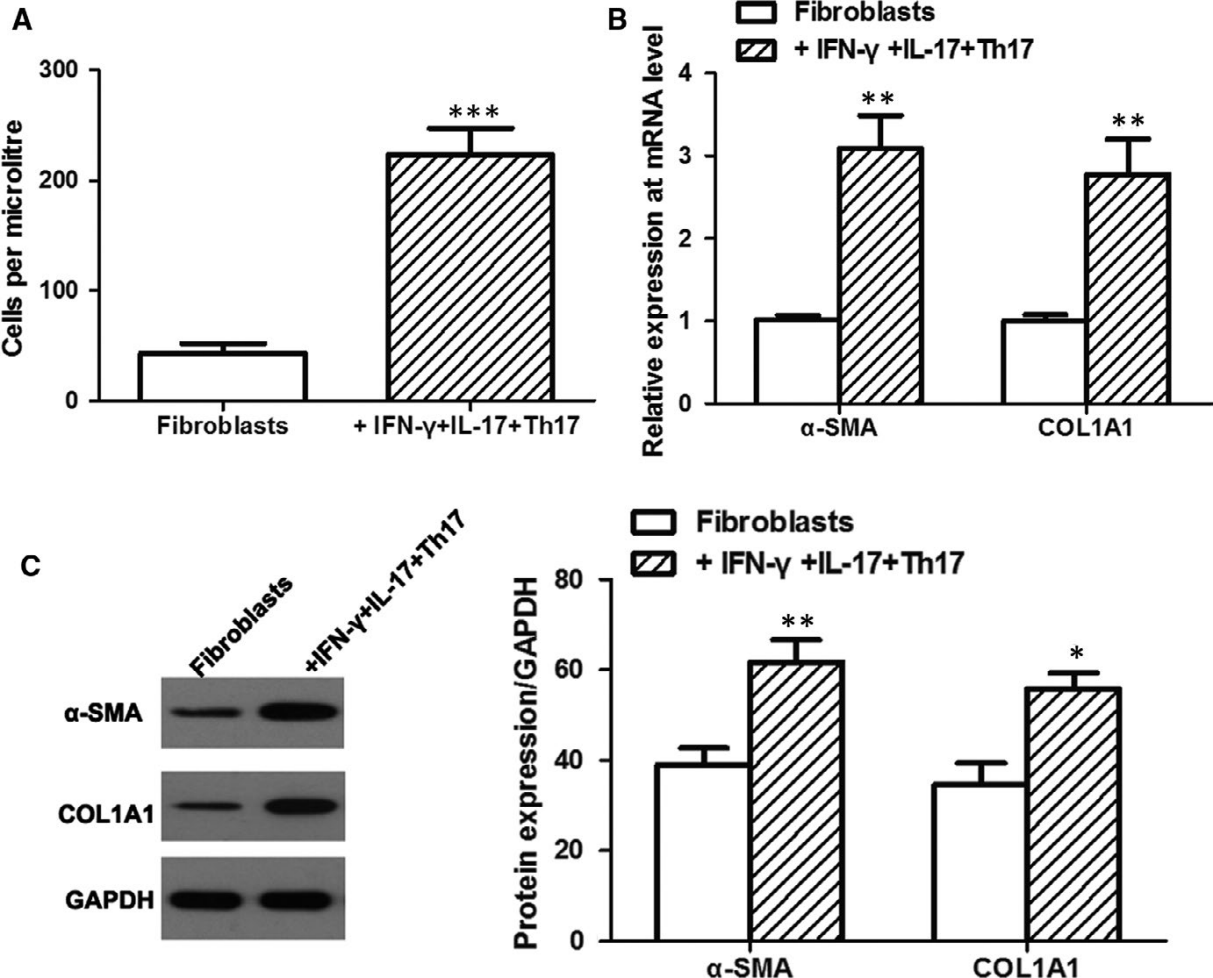
The co‐culture with IFN‐γ^+^IL‐17^+^Th17 cells significantly increased the number of fibroblasts in vitro (A). Moreover, the expressions of α‐SMA and COL1A1 were obviously up‐regulated at both mRNA (B) and protein (C) levels after co‐culture. *P < .05, **P < .01, ***P < .001

Dermal fibroblasts were isolated from SSc skin tissues and cultured in vitro in our study. We co‐cultured dermal fibroblasts with IFN‐γ^+^IL‐17^+^Th17 cells for 3 days and performed immunohistochemistry to investigate the number of dermal fibroblasts which were immunolabelled by α‐SMA. Fibroblasts cultured alone were employed as controls. Compared to control group, the number of positively stained cells of α‐SMA was significantly increased in co‐culture groups (*P* < .05 for all) (Figure 5A). Collagen‐secreting capacity of fibroblasts was estimated through COL1A1 protein. Western blot analysis was adopted to investigate the expressions of α‐SMA and COL1A1. Three days after co‐culture, the cells and culture supernatants were harvested for qRT‐PCR and Western blot analyses. As displayed in Figure 5B,C, the expressions of α‐SMA and COL1A1 were obviously up‐regulated at both mRNA and protein levels, revealing increased fibroblast number and enhanced collagen‐secreting capacity.

### 3.7 IFN‐γ^+^IL‐17^+^Th17 cells contributed to fibrosis through producing IL‐21 in SSc

**FIGURE 6:**
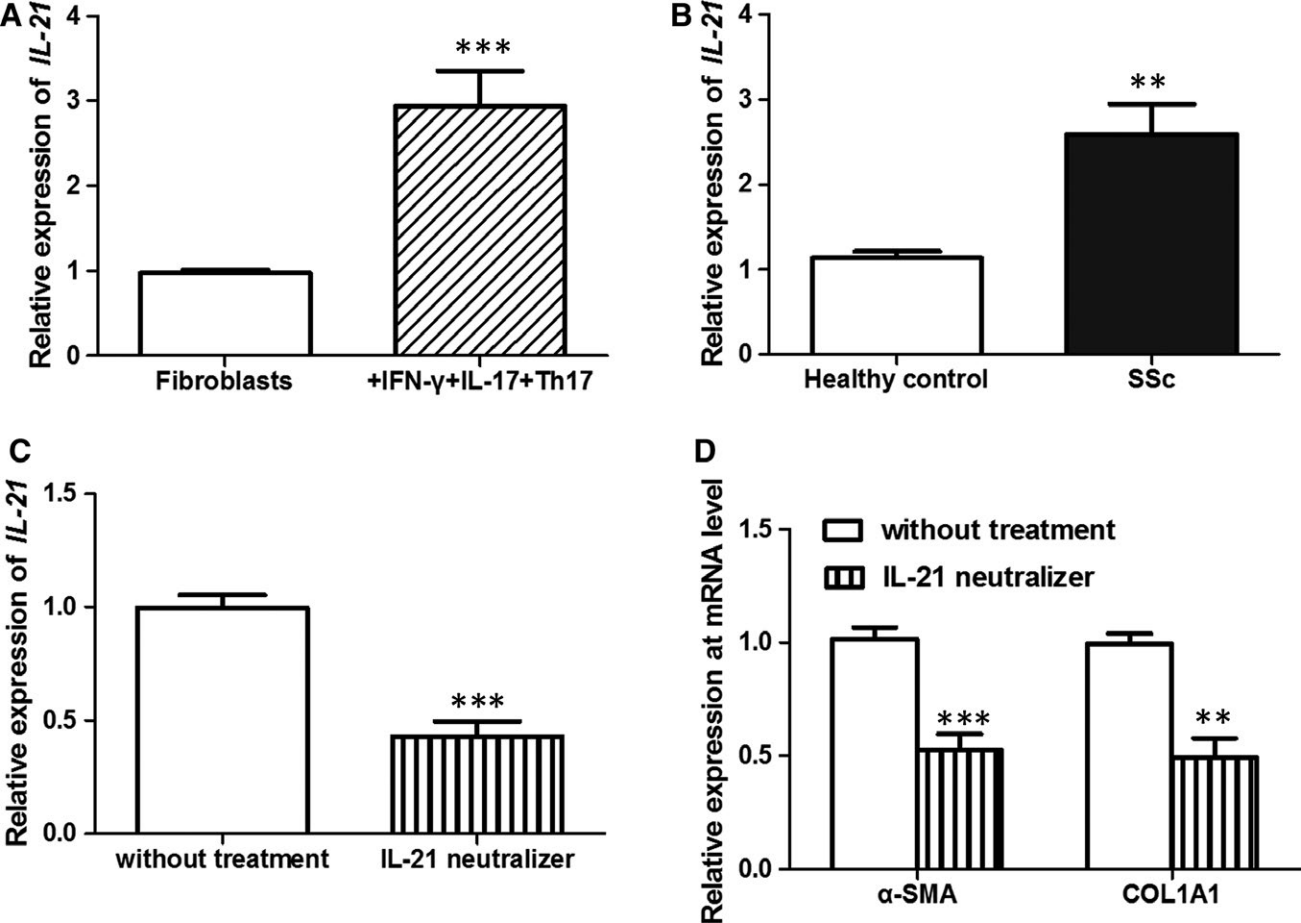
IL‐21 mRNA level was significantly increased in the co‐culture medium of fibroblasts and IFN‐γ^+^IL‐17^+^Th17 cells (A). Moreover, compared to healthy skin, SSc skin also showed up‐regulated IL‐21 mRNA (B). The treatment with IL‐21 neutralizer could reduce the level of IL‐21 mRNA in co‐culture medium (C). In addition, IL‐21 neutralizer treatment resulted in the down‐regulation of α‐SMA and COL1A1 mRNAs (D). **P < .01, ***P < .001

To explore mechanisms underlying the function of IFN‐γ^+^IL‐17^+^Th17 cells on fibrosis, we investigated cytokine concentration in co‐culture medium. In our preliminary experiments, we found that the level of IL‐21 was highest in co‐culture medium. Moreover, published articles had demonstrated that IL‐21 might induce CD4^+^ T cell differentiation towards Th17 cells^22^ and that it also wielded pro‐fibrogenic function.^23^ Therefore, IL‐21 was considered as an effector in our current study. QRT‐PCR analysis demonstrated that the mRNA level of IL‐21 was significantly increased in co‐culture medium of IFN‐γ^+^IL‐17^+^Th17 cells and fibroblasts (*P* < .001) (Figure 6A). Moreover, compared to healthy ones, SSc skin specimens showed remarkable up‐regulation of IL‐21 mRNA (*P* < .01) (Figure 6B).

In addition, to further confirm the function of IL‐21 on fibrosis progression mediated by IFN‐γ^+^IL‐17^+^Th17 cells, IFN‐γ^+^IL‐17^+^Th17 cells were pre‐treated with neutralizing anti‐IL‐21 antibody before co‐culture. Then, 3 days after co‐culture, we found that the mRNA level of IL‐21 was decreased, in comparison with cells without pre‐treatment (*P* < .001) (Figure 6C). Moreover, the levels of α‐SMA and COL1A1 mRNAs also exhibited a decreasing tendency (*P* < .01) (Figure 6D). All data suggested that IL‐21 secreted by IFN‐γ^+^IL‐17^+^Th17 cells played an important role in fibrosis in SSc.

## 4 DISCUSSION

SSc is a fatal disease and exhibits the accumulation of collagen in skin and internal organs, thus resulting in multiple organ fibrosis and high death rate.^24^ According to the involvement of organs, multiple anti‐fibrosis treatments have been developed for SSc patients, but their efficacy is unsatisfactory.^25^ Because of the unclear aetiology of this disease, it remains a great challenge to identify key triggering events which drive fibrosis progression in SSc. It is generally accepted that the dysregulation of immune system is closely correlated with the progression of SSc.^26^ Th17 cells could produce pro‐inflammatory cytokines and play important roles in autoimmune diseases, like SSc.^27^ In our current study, we explored the function of IFN‐γ^+^IL‐17^+^Th17 cells in SSc. The percentage of IFN‐γ^+^IL‐17^+^Th17 cells was significantly increased in SSc blood and tissue specimens; moreover, such elevated level showed positive association with high disease activity scores, long disease duration and poor skin scores. The activation of IFN‐γ^+^IL‐17^+^Th17 cells might promote the proliferation of fibroblasts and enhance their collagen‐secreting capacity. The pro‐fibrogenic function of IFN‐γ^+^IL‐17^+^Th17 cells was mediated by IL‐21 in SSc.

IFN‐γ^+^IL‐17^+^Th17 cells are a subgroup of Th17 cells, and they can secrete both IL‐17 and IFN‐γ. IFN‐γ is a cytokine typically produced by Th1 cells. However, because of their high plasticity, Th17 cells may differentiate into Th1‐like phenotype under certain inflammation conditions, thus producing IFN‐γ.^16^ IFN‐γ^+^IL‐17^+^Th17 cells have been reported to be implicated in several autoimmune disorders, such as sarcoidosis^16^ and coronary artery atherosclerosis.^28^ However, the distribution of IFN‐γ^+^IL‐17^+^Th17 cells has been rarely reported. In this study, we found that the percentage of IFN‐γ^+^IL‐17^+^Th17 cells was significantly higher in SSc blood and skin specimens than in healthy controls. Moreover, the levels of IFN‐γ and IL‐17 were obviously increased in SSc, compared to healthy controls. In addition, we also found that SSc patients with high percentage of IFN‐γ^+^IL‐17^+^Th17 cells were more likely to face late stages, longer disease duration, high disease activity and poor skin scores. All clinical results demonstrated that excess activation of IFN‐γ^+^IL‐17^+^Th17 cells might be a risk factor for SSc and predict aggressive disease progression for the patients. The present study explored the presence of IFN‐γ^+^IL‐17^+^Th17 cells in SSc and their close association with disease activity.

To further explore potential mechanisms of IFN‐γ^+^IL‐17^+^Th17 cells functioning in SSc, we co‐cultured IFN‐γ^+^IL‐17^+^Th17 cells and dermal fibroblasts isolated from SSc skin. Three days later, we found that the number of fibroblasts was significantly increased, and the levels of COL1A1 also showed an increasing tendency. The results might reveal that IFN‐γ^+^IL‐17^+^Th17 cells promoted fibroblasts’ proliferation and enhanced their collagen‐secreting ability, thus contributing to fibrosis. The pro‐fibrogenic function of Th17 cells had been reported in several published articles. For instance, increased levels of Th17 cells were observed in renal ischaemia/reperfusion (I/R) rat combined with lung fibrosis, and suppressing Th17 cell infiltration could reduce fibrosis.^29^ In this study, we proved the pro‐fibrogenic ability of IFN‐γ^+^IL‐17^+^Th17 cells. The pro‐fibrogenic ability of IL‐17 cytokine was reported in several published articles.^30^, ^31^, ^32^ For example, Latella et al reported that the presence of Th17/IL‐17 was involved in intestinal fibrosis, and the application of anti‐IL‐17 antibody might significantly alleviate 2,4,6‐trinitrobenzene sulphonic acid (TNBS)‐induced colorectal fibrosis in mice.^30^ In addition to colorectal fibrosis, IL‐17 was also correlated with liver fibrosis,^31^ skin fibrosis,^32^ etc Combined with our results, it could be reasonably inferred that IL‐17 held pro‐fibrotic capacity.

Th17 cells hold high plasticity, and they could produce diverse cytokines under different inflammation conditions.^33^ Lei et al reported that with the presence of IL‐21 in SSc mouse model, CD4^+^ cells could differentiate into Th17 cells.^22^ Moreover, increased levels of IL‐21 produced by Th17 cells contribute to fibrosis process.^23^ Thus, we have been suggested that IL‐21 might act as an effector of IFN‐γ^+^IL‐17^+^Th17 cells in fibrosis process. In our study, we found that the levels of IL‐21 were obviously increased in co‐culture cell medium which contained IFN‐γ^+^IL‐17^+^Th17 cells and fibroblasts. Moreover, treatment with IL‐21 neutralizer resulted in the down‐regulation of α‐SMA and COL1A1, revealing reduced fibroblasts’ proliferation and attenuated collagen‐secreting ability. Ricard et al reported that enhanced production of IL‐21 by circulating follicular helper T cells could also contribute to SSc progression.^34^ IL‐21 pathway might be involved in the process of SSc. In addition to IFN‐γ and IL‐17, IFN‐γ^+^IL‐17^+^Th17 cells might also produce IL‐21 to promote SSc severity. Immune suppression strategy specific to IFN‐γ^+^IL‐17^+^Th17 cells might be an effective approach for the treatment of SSc.

Several limitations in our study should be stated. Firstly, the relatively small sample size might reduce statistical power of our clinical analyses. Moreover, because of poor prognosis in males, unequal number of male and female cases might cause bias into final results. Secondly, because of long disease duration, treatment strategies for included patients were unclear, which might influence our results. Thirdly, Th17 cells could promote the proliferation of fibroblasts, produce multiple cytokines and modify inflammation microenvironment, thus contributing to fibrosis process.^14^ In addition to IL‐21, IFN‐γ^+^IL‐17^+^Th17 cells might produce other cytokines to promote fibrosis, but relevant factors were not explored in our study. Additionally, in vivo experiments are required to verify our results.

In conclusion, the distribution of IFN‐γ^+^IL‐17^+^Th17 cells is significantly increased in SSc cases and shows positive association with high disease activity score. In vitro, IFN‐γ^+^IL‐17^+^Th17 cells could promote the proliferation of fibroblasts and enhance their collagen‐secreting ability. IFN‐γ^+^IL‐17^+^Th17 cells may contribute to fibrosis through IL‐21 pathway in SSC.

## CONFLICT OF INTEREST

None.

## AUTHOR CONTRIBUTIONS

XX conceived and designed the experiments; AL conceived and performed the experiments; YZ prepared figures. HZ wrote the main manuscript text. All authors reviewed the manuscript. With the approval of First Medical Center of Chinese PLA General Hospital Ethics Committee, written informed consent was obtained from every patient.

## Data Availability

The data used to support the findings of this study are available from the corresponding author upon request.
